# Supplementary figures and images for: Natural allelic variations of Saccharomyces cerevisiae impact stuck fermentation due to the combined effect of ethanol and temperature; a QTL-mapping study
Source: BMC Genomics. 2019 Aug 28;20:680. doi: 10.1186/s12864-019-5959-8 (PMC6714461; doi:10.1186/s12864-019-5959-8)

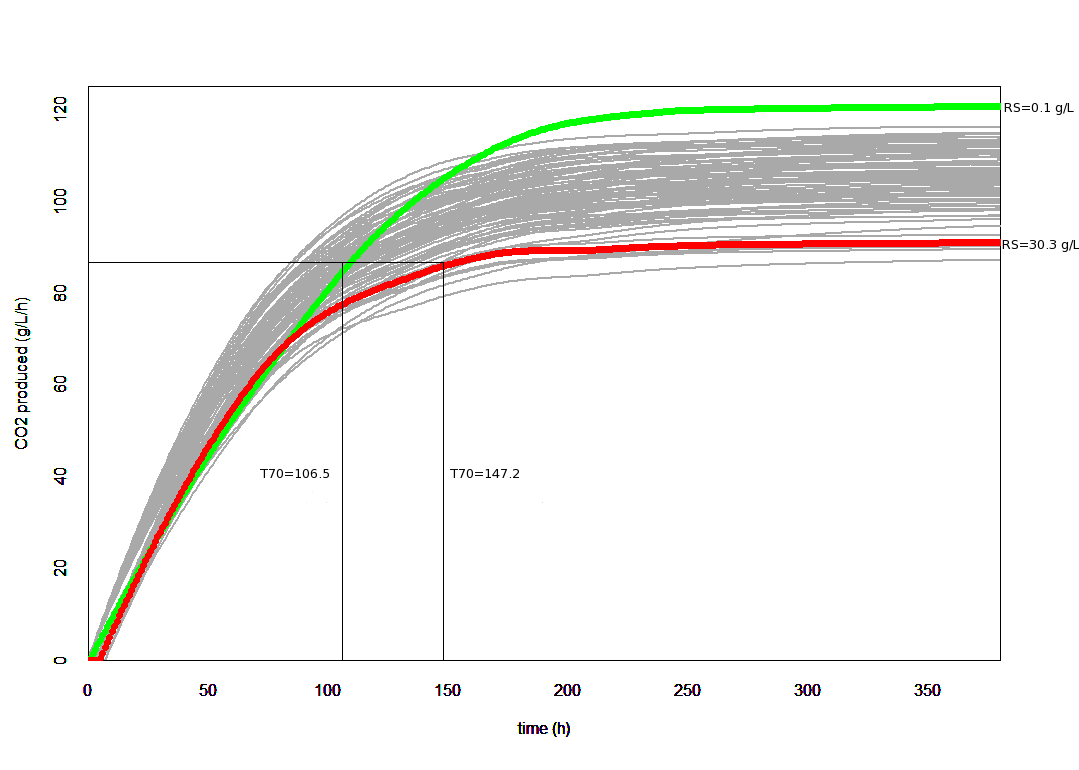

Supplement: Supplementary file 1 — Fermentation kinetics of the 77 segregants and the two parental strains G-4A (red) and B-1A (green). (TIFF 2437 kb) [file 12864_2019_5959_MOESM1_ESM.tiff]

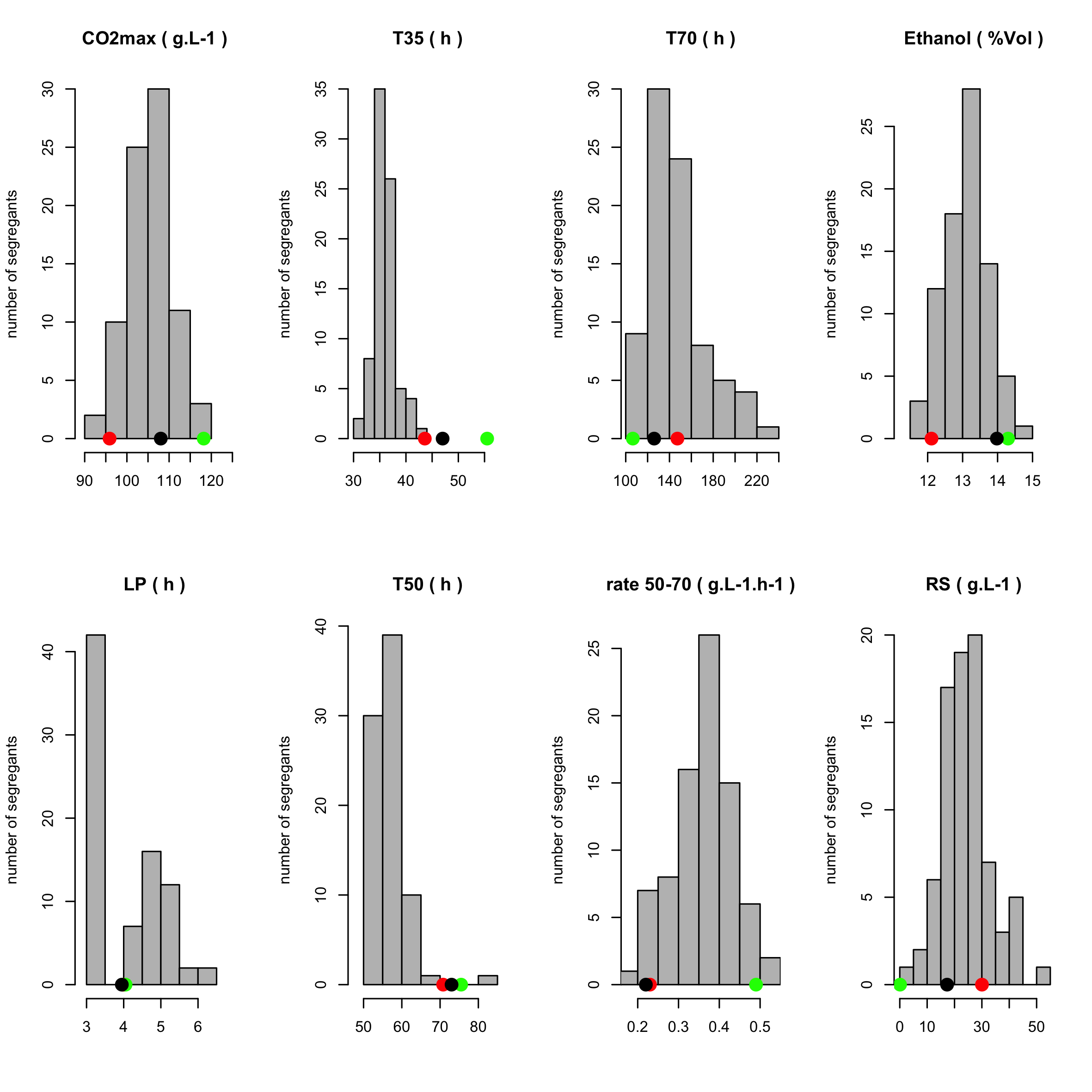

Supplement: Supplementary file 2 — Trait distribution among H4 progeny. The green, red and black full dots represent the parental values of the strains B-1A, G-4A and H4. (TIFF 21796 kb) [file 12864_2019_5959_MOESM2_ESM.tiff]

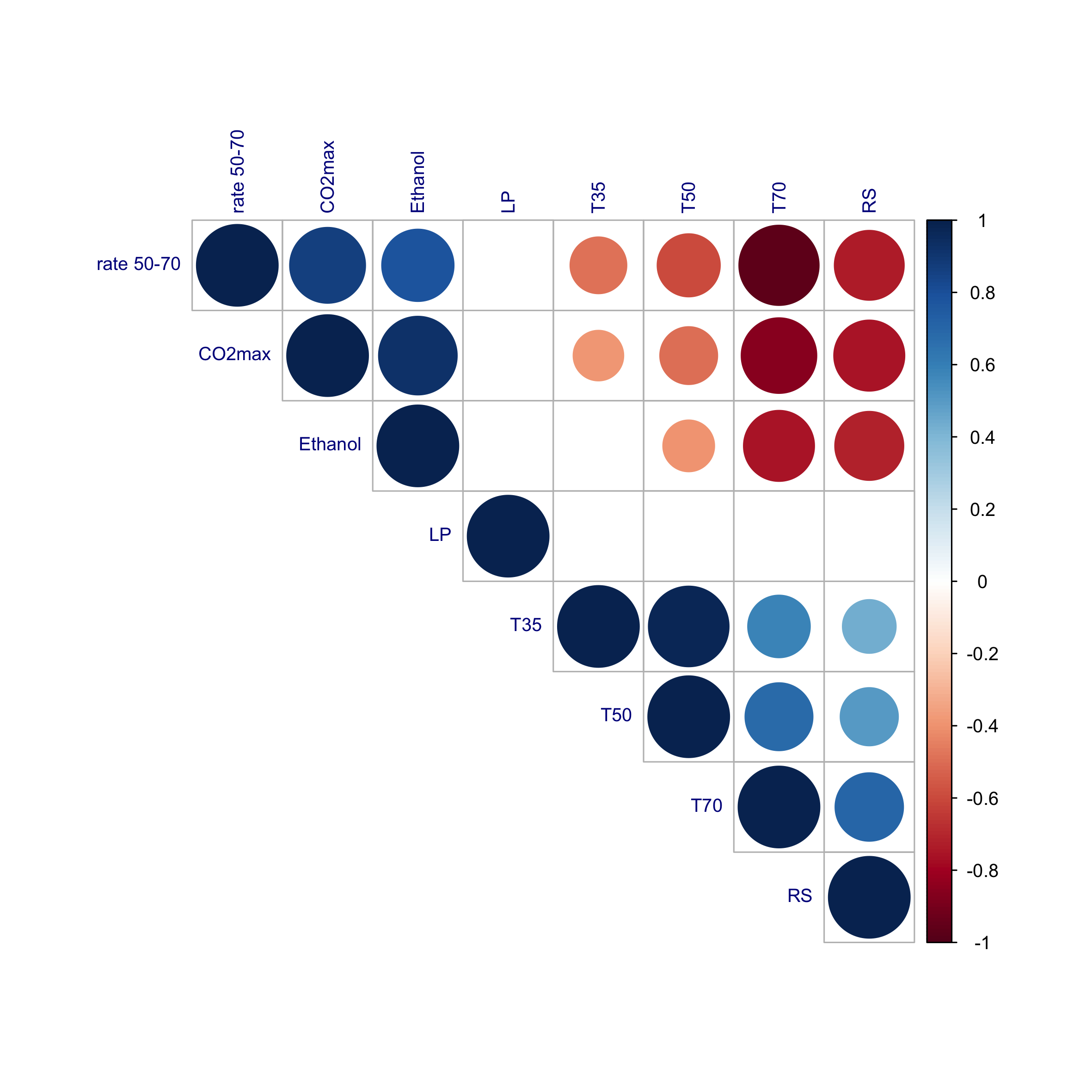

Supplement: Supplementary file 3 — Correlation analysis within each trait investigated in the H4 progeny. The test applied was the Pearson test. The size and the color of the dots represent the pvalue and the correlation rate, respectively. Only significant correlations corrected p values (BH) lower than 0.001 were shown. (TIFF 21796 kb) [file 12864_2019_5959_MOESM3_ESM.tiff]

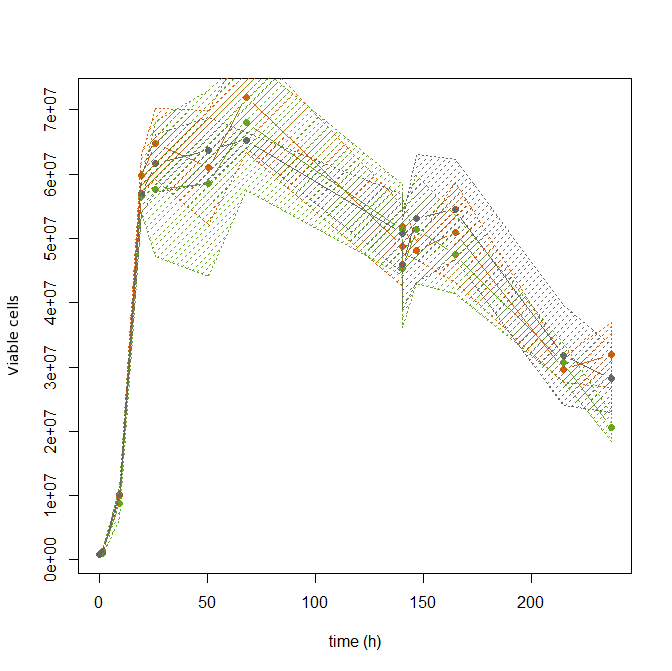

Supplement: Supplementary file 7 — Biomass viability for the hemyzygous hybrids ΔOYE2B::KanMx4/OYE2G (red) and OYE2B/ΔOYE2G::KanMx4 (green) and H4 (black). The dots represent mean value for the sampling points and the shaded area the standard deviation estimated with at least five repetitions. (TIFF 1720 kb) [file 12864_2019_5959_MOESM7_ESM.tiff]

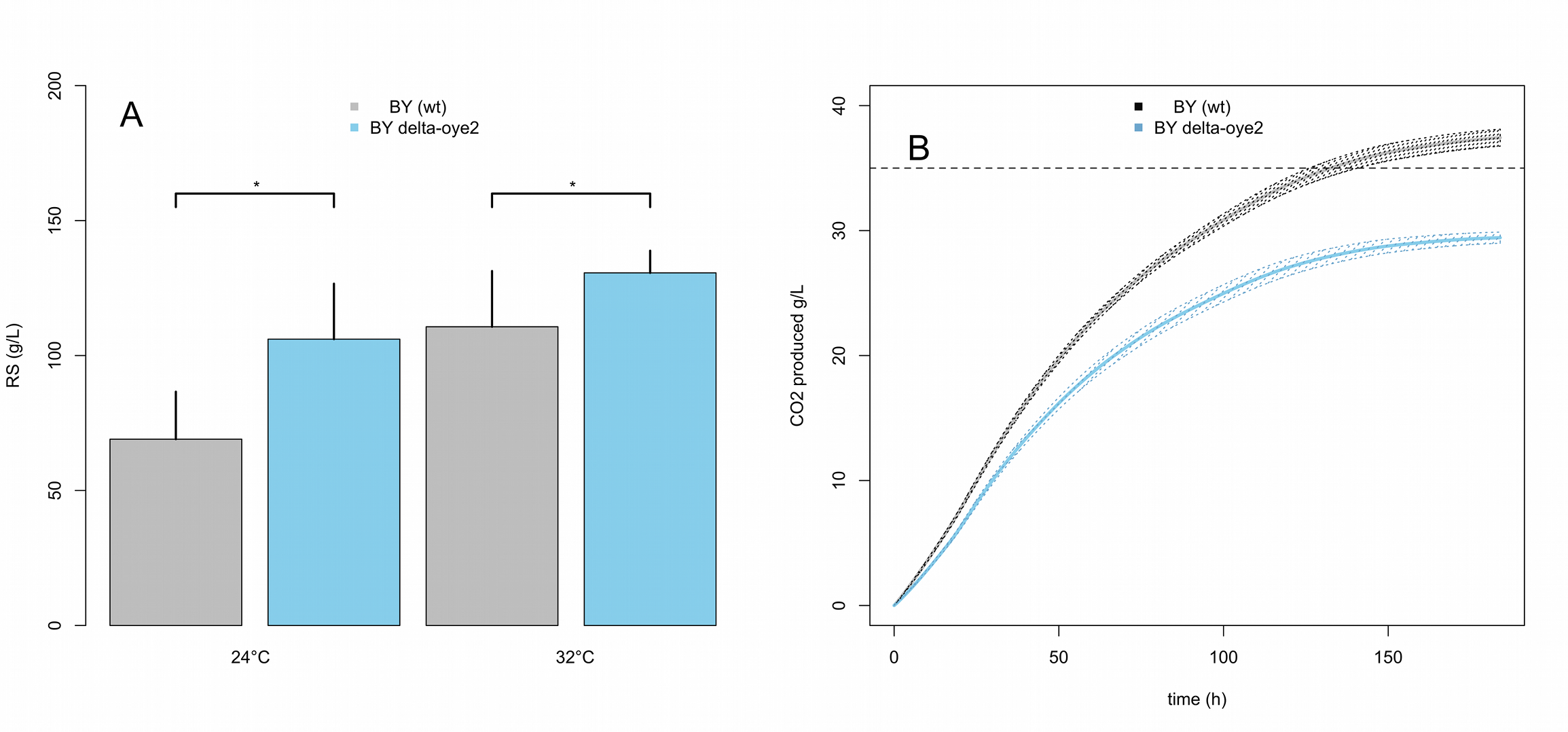

Supplement: Supplementary file 8 — Panel A. The bar plots represent the average values of residual sugars after isothermal fermentations carried out at 24, and 32 °C in the laboratory strain background (BY4741). The genotypes Δoye2 and OYE2(wt) were shown in blue and grey, respectively. Bars represent standard error of five repetitions, the statistical differences between the hemizygous was tested by a Wilcoxon-Mann-Whitney Test (the p value is coded as follow, ‘*’ = p < 0.05). Panel B. Fermentation kinetics (CO2 produced time course at 24 °C.) for the same strains and with the same color key (TIFF 9490 kb) [file 12864_2019_5959_MOESM8_ESM.tif]

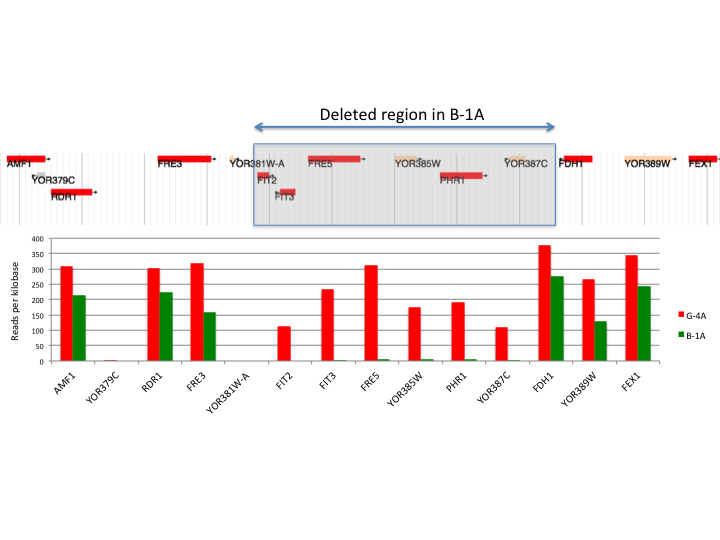

Supplement: Supplementary file 9 — This figure illustrates the deletion observed in the strain B-1A for the genomic region encompassing the genes FIT2, FIT3, FRE5, YOR385W, PHR1, YOR387C. The deletion was found by comparing the read per kb observed for all the genes of the right arm of chromosome XV. Green bars (B-1A), red bars (G-4A). (TIFF 1480 kb) [file 12864_2019_5959_MOESM9_ESM.tiff]

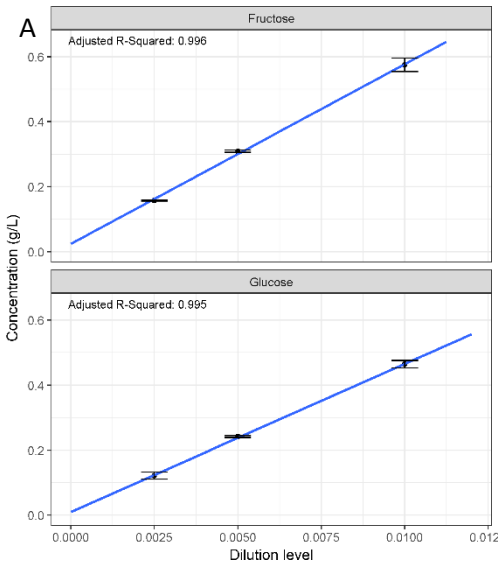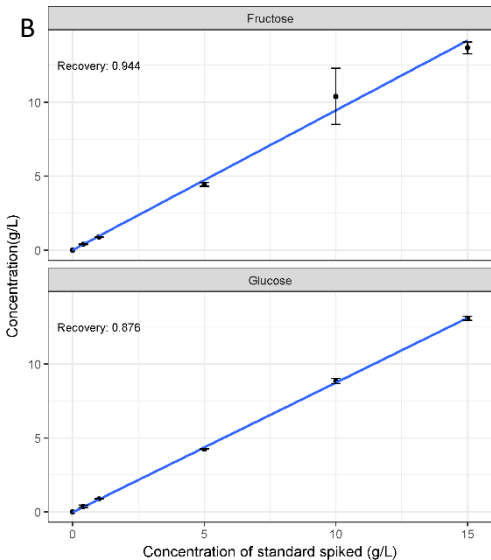

Supplement: Supplementary file 10 — Reliability of the enzymatic assay of glucose and fructosePanel A. Linearity of the assay. The values shown are the average of two repetitions and the error bar indicates the standard deviation. The blue line indicates the linear regression line, the adjusted R-Squared is indicated. Panel B. Recovery of the assay. Each point represents the average value of three repetitions. The error bars indicate the standard deviation. The blue line indicates the linear regression line. Recovery is indicated. Linearity of the assay (panel A). The concentration of a sample was measured at different dilution levels (1/100, 1/200 and 1/400). Linear regression with a R-Squared close to 1 indicates the linearity of the enzyme assay in this range (0.15 g/L - 0.6 g/L). Recovery of the assay (panel B). Different concentrations of glucose or fructose are added to a sample (0.4 g/L, 1 g/L, 10 g/L and 15 g/L). The slope of the linear regression line indicates which part of the added concentration is actually measured (recovery). A slope close to 1 shows a good recovery of the assay between 0.4 g/l and 15 g/l. (PDF 75 kb) [file 12864_2019_5959_MOESM10_ESM.pdf]
